# Identification of cardiomyopathy-related core genes through human metabolic networks and expression data

**DOI:** 10.1186/s12864-021-08271-0

**Published:** 2022-01-12

**Authors:** Zherou Rong, Hongwei Chen, Zihan Zhang, Yue Zhang, Luanfeng Ge, Zhengyu Lv, Yuqing Zou, Junjie Lv, Yuehan He, Wan Li, Lina Chen

**Affiliations:** College of Bioinformatics Science and Technology, Harbin Medical University, Harbin, Heilongjiang China

**Keywords:** Cardiomyopathy, Human metabolic network, Expression data, Module, Core genes

## Abstract

**Background:**

Cardiomyopathy is a complex type of myocardial disease, and its incidence has increased significantly in recent years. Dilated cardiomyopathy (DCM) and ischemic cardiomyopathy (ICM) are two common and indistinguishable types of cardiomyopathy.

**Results:**

Here, a systematic multi-omics integration approach was proposed to identify cardiomyopathy-related core genes that could distinguish normal, DCM and ICM samples using cardiomyopathy expression profile data based on a human metabolic network. First, according to the differentially expressed genes between different states (DCM/ICM and normal, or DCM and ICM) of samples, three sets of initial modules were obtained from the human metabolic network. Two permutation tests were used to evaluate the significance of the Pearson correlation coefficient difference score of the initial modules, and three candidate modules were screened out. Then, a cardiomyopathy risk module that was significantly related to DCM and ICM was determined according to the significance of the module score based on Markov random field. Finally, based on the shortest path between cardiomyopathy known genes, 13 core genes related to cardiomyopathy were identified. These core genes were enriched in pathways and functions significantly related to cardiomyopathy and could distinguish between samples of different states.

**Conclusion:**

The identified core genes might serve as potential biomarkers of cardiomyopathy. This research will contribute to identifying potential biomarkers of cardiomyopathy and to distinguishing different types of cardiomyopathy.

## Introduction

Cardiomyopathy is a disease of the heart muscle with major abnormalities in the structure and function of the heart, and cause the myocardium to become weak and ineffective [1]. The World Health Organization separates the various cardiomyopathies into several types based on the main pathophysiology and etiology/pathogenic factors. Dilated cardiomyopathy (DCM) and ischemic cardiomyopathy (ICM) are two major types with essentially different etiology. DCM could be caused by viral infections, autoimmunity, and genetic factors, while ICM was mainly caused by long-term myocardial ischemia due to atherosclerotic lesions. Moreover, DCM and ICM often exhibit similar clinical symptoms [2–4], making them two highly related pathologies that have not been fully characterized. Therefore, effective differentiation between DCM and ICM is of great importance in preventing and personalizing the treatment of cardiomyopathy in patients. Giraldo et al. used respiratory sinus arrhythmia (RSA) index of the parasympathetic system quantified by linear and non-linear analysis methods to discriminate between DCM and ICM with high sensitivity and specificity [5]. Sweet et al. used differentially expressed genes (DEGs) and pathway analysis to identify DEG signatures that could correctly classify the phenotypes of ICM and DCM samples [6].

In addition to gene expression, abnormal metabolism can also lead to poor heart function, which can affect the functions of myocardial cell [7]. Zhao et al. evaluated plasma metabolomics of patients with DCM or ICM through comprehensive metabolomic analysis to identify plasma metabolite biomarkers [8]. And some studies have shown that changes in myocardial metabolism are one of the important pathogenic factors of diabetic cardiomyopathy [9]. Metabolic networks can reflect a variety of chemical reactions catalyzed by gene-encoded enzymes and their interaction systems. Wang et al. constructed a lipid metabolism network and identified lipid subnetworks and clusters that involved in the pathogenesis of cardiovascular diseases [10]. Moreover, the identification of genes related to diseases in networks through shortest path analysis has been widely used to study the mechanism of diseases. Yang et al. performed shortest path analysis to explore the key drug targets of LianXia NingXin formulations for the treatment of coronary heart disease-related phenotypes (e.g., co-morbid diseases and symptoms) [11].

**Fig. 1:**
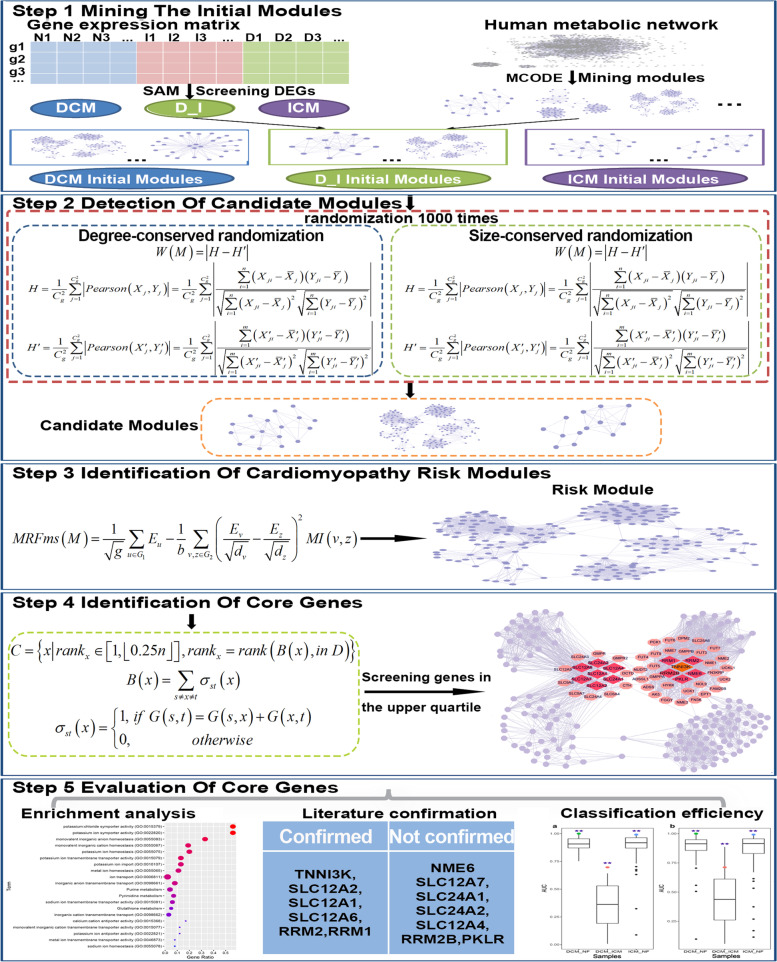
Flow chart of this study. Step 1: mining the initial modules from the network modules mined by MCODE according to DEGs. Step 2: detection of candidate modules based on the significance of the Pearson correlation coefficient difference score of the initial modules. Step 3: identification of the cardiomyopathy risk modules significantly related to DCM and ICM according to the importance of the module score based on Markov random field. Step 4: identification of core genes from the cardiomyopathy risk module based on the shortest path between known disease-causing genes. Step 5: evaluation of core genes from three aspects: enrichment analysis, literature confirmation and classification efficiency

Therefore, in this study, a systematic multi-omics integration approach was proposed to identify cardiomyopathy-related core genes based on metabolic networks and expression data. First, the Significant Analysis of Microarray (SAM) method was used to screen DEGs between samples of different states in the expression data, and three sets of initial modules containing DEGs were obtained from the modules mined by Molecular Complexity Detection (MCODE). Two permutation tests were used to evaluate the significance of the Pearson correlation coefficient difference score of the initial modules, and candidate modules were screened out. Then, according to the significance of the module score based on Markov random field (MRF), the cardiomyopathy risk module that was significantly related to DCM and ICM was determined. Finally, based on the shortest path between known genes, 13 core genes closely related to cardiomyopathy were identified (Fig. 1). Our method provided valuable ideas for identifying potential cardiomyopathy genes that could effectively distinguish different types of cardiomyopathy.

## Results

### Initial and candidate modules

From the reconstructed metabolic network, modules with nodes ≥4 were selected. A total of 52 modules were identified from the reconstructed metabolic network using MCODE (see Materials and Methods for details). Of these modules, 8 modules containing DEGs of DCM and ICM samples (DCM_ICM was used to indicate the two states of DCM and ICM.) were screened as initial D_I modules, 21 modules containing DEGs of DCM and normal samples (DCM_NF was used to indicate the two states of DCM and normal.) were screened as initial DCM modules (containing seven initial D_I modules), and 37 modules containing DEGs of ICM and normal samples (ICM_NF was used to indicate the two states of ICM and normal.) were screened as initial ICM modules (containing 8 initial D_I modules and 21 initial DCM modules).

Two permutation tests of Pearson difference scores were performed on all initial modules, and finally 3 modules that were significantly different compared with random modules of the same degree and the same scale were selected as candidate modules (both *P* values < 0.05), including 2 ICM modules (ICM-module1, ICM-module2), 1 DCM module (DCM-module1) and 1 D_I module (D_I-module1). Among them, DCM-module1 and D_I-module1 were the same module.

The expression values of all genes in the three candidate modules were used as classification features to classify samples of different states (DCM_NF, ICM_NF or DCM_ICM), respectively. The genes of the three modules had good classification performance for normal and disease samples. However, for the classification of ICM and DCM samples, the three modules showed different results. Among them, the DCM-module1/D_I-module1 (containing 4 DCM_ICM DEGs) could effectively distinguish between DCM_ICM samples, while the modules ICM-module1 and ICM-module2 could not (Table 1).

**Table 1:** Classification performance of candidate modules

Modules	AUC for normal and disease samples	AUC for ICM and DCM samples
ICM-module1	0.93	0.31
ICM-module2	0.79	0.40
DCM-module1/D_I-module1	0.99	0.71

### Cardiomyopathy risk modules

The module scores based on MRF (MRFmss) for 3 candidate modules were calculated (see Materials and Methods for details) [12, 13], and compared with that of random modules. One cardiomyopathy risk module, D_I-module1, containing 205 genes, with significantly higher MRFms was identified (*p* < 0.05).

**Fig. 2:**
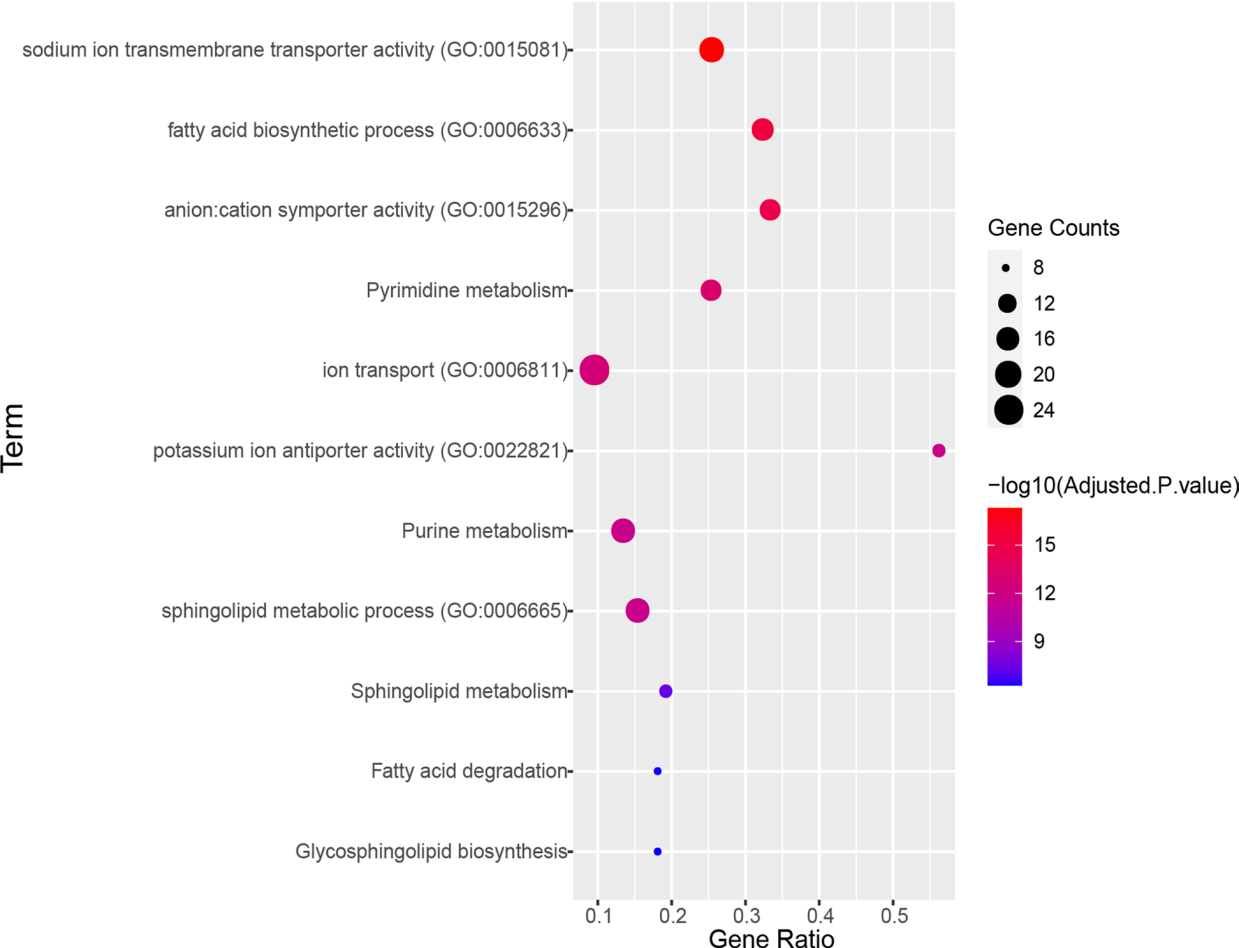
Some pathways and functions enriched by the cardiomyopathy risk module

This cardiomyopathy risk module was significantly enriched in pathways and functions (see Materials and Methods for details) related to cardiomyopathy (some are in Fig. 2).

**Fig. 3:**
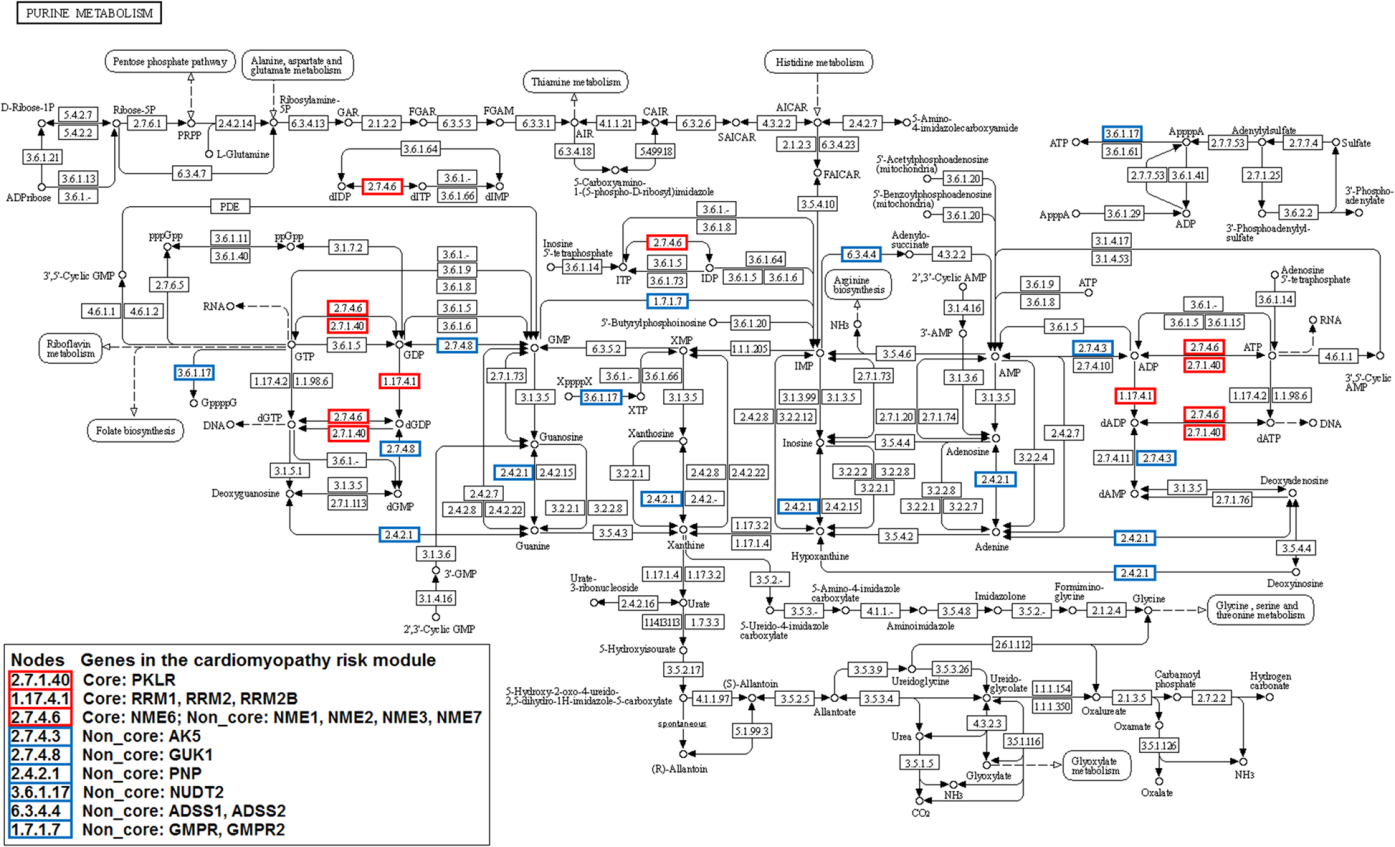
Purine metabolism pathway [16]. The red rectangles indicate the core genes in the cardiomyopathy risk module, and the blue ones indicate the non-core genes

“Purine metabolism” and “Pyrimidine metabolism” are two pathways closely related to nucleotide content. Studies in human and animal models have demonstrated that many disorders of purine and pyrimidine nucleotide content in the myocardium played a role in the pathogenesis of muscle dysfunction in diseases such as coronary heart disease and left ventricular hypertrophy [14, 15]. Genes of the cardiomyopathy risk module were widely distributed in both pathways, suggesting the involvement of the cardiomyopathy risk module in muscle dysfunction of the myocardium diseases. For example, in the “Purine metabolism” pathway, the cardiomyopathy risk module genes mainly encode various enzymes involved in energy conversion in the pathway, including various kinases, reductases, hydrolases and synthases (Fig. 3). They are mainly involved in the pathway module Adenine ribonucleotide biosynthesis and Guanine ribonucleotide biosynthesis. These enzymes catalyze the hydrolysis of tetraphosphate to produce ATP, and the mutual conversion of ATP/GTP and ADP/GDP.

Functions including “ion transport”, “anion: cation symporter activity”, “potassium ion antiporter activity”, “sodium ion transmembrane transporter activity” were related to ion transport in cells. Through a series of activities of diverse ion channels, the excitability of cardiac myocytes is caused by ionic fluxes [17, 18]. The “Fatty acid degradation” pathway and “fatty acid biosynthetic process” function were related to fatty acids. Fatty acids were the main energy substrates of the heart, provided energy for myocardial contraction, and were essential substrates for the synthesis of sphingolipids [19, 20]. Sphingolipids regulated many cellular processes that occurred in primary and secondary cardiomyopathy, and were also involved in functional categories and pathways, such as “Glycosphingolipid biosynthesis”, “Sphingolipid metabolism” and “sphingolipid metabolic process “. And a large number of studies have shown that disorders of sphingolipid metabolism can cause changes in the structure and function of cardiomyocytes [21–23].

### Cardiomyopathy-related core genes

**Fig. 4:**
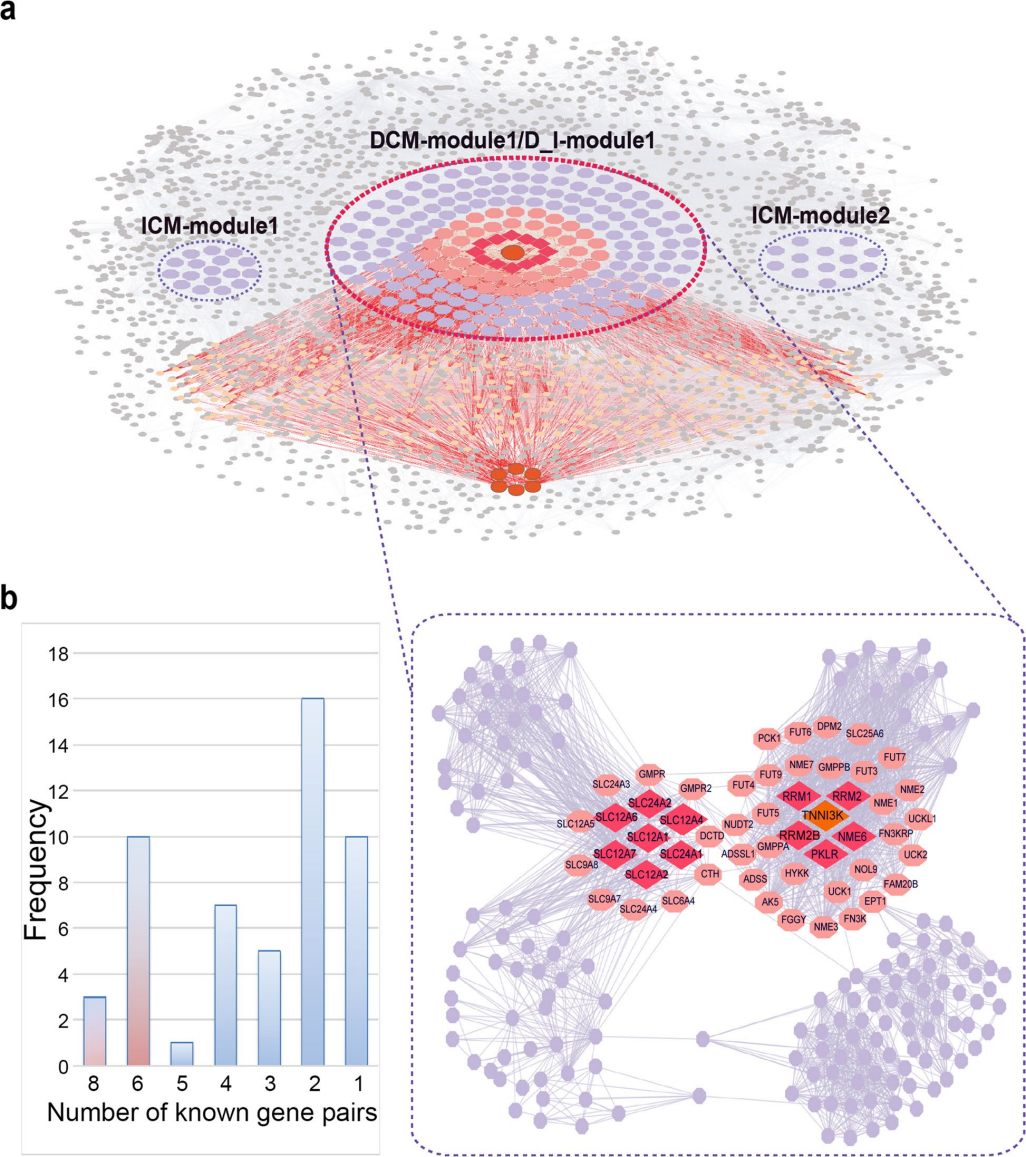
Identification of core genes. **a** The overall reconstructed metabolic network. Genes in the red dashed circle comprised the cardiomyopathy risk module. Nodes in light orange are the genes on the shortest paths between known genes. The red lines are the shortest paths between known genes. **b** The picture on the left is the distribution of the number of known gene pairs linked by candidate genes via the shortest paths, and the red bars are the top 25%. The picture on the right is the enlarged picture of the cardiomyopathy risk module. Nodes in purple are the genes in the module, in light red are the candidate genes, in dark red and orange diamonds are the core genes. The dark orange ones are known genes of cardiomyopathy

In the cardiomyopathy risk module, 52 candidate genes were located on the shortest paths between known genes. For each candidate gene, the number of known gene pairs linked by it via the shortest paths was calculated. Finally, 13 genes linked more than 6 (the top quartile) known gene pairs were identified as cardiomyopathy-related core genes of the module (containing 2 ICM_NF DEGs, 1 DCM_NF DEG, and 1 DCM_ICM DEG and 1 known pathogenic gene) (Fig. 4).

The relationship between core genes and cardiomyopathy was analyzed from the following aspects.

#### Functional enrichment analysis

**Fig. 5:**
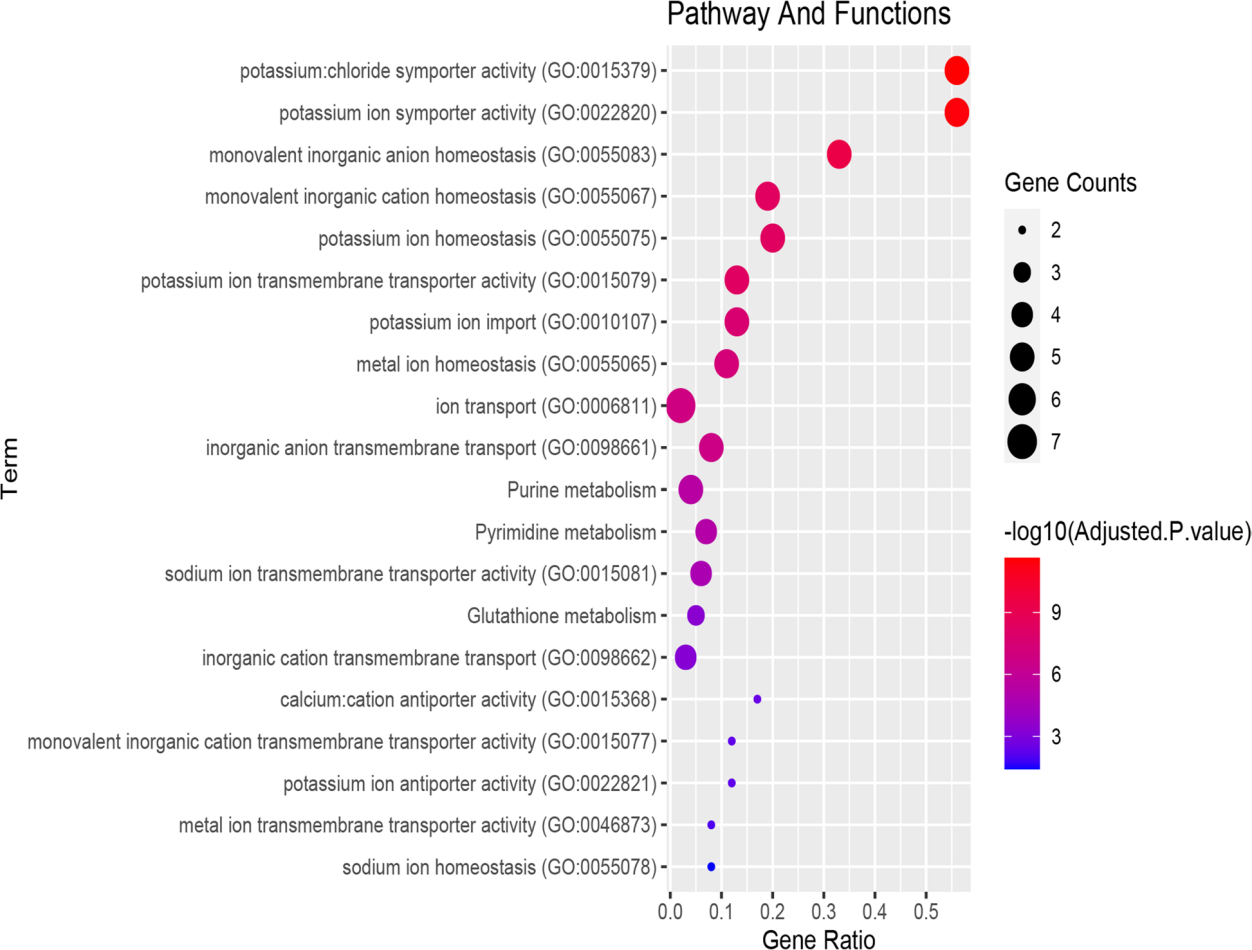
Enriched functions and pathways of core genes

In order to understand the relationship between core genes and diseases, a functional enrichment analysis of core genes was performed. These core genes were significantly enriched in multiple Gene Ontology (GO) functions and Kyoto Encyclopedia of Genes and Genomes (KEGG) pathways associated with cardiomyopathy and related diseases or tissues (FDR adjusted *p* < 0.05) (Fig. 5), including some functions and pathways enriched by the cardiomyopathy risk module, such as “Purine metabolism” pathway (Fig. 3) and other pathways and functional categories. In the “Purine metabolism” pathway, the core genes encode two kinases and one reductase. Two kinases are involved in the hydrolysis and synthesis of the energy substance ATP, while reductase is responsible for the de novo conversion of ribonucleoside diphosphates to deoxyribonucleoside diphosphates, and two kinases and reductase are involved in subsequent DNA synthesis.

The homeostasis of Glutathione (GSH), which could be affected by the “Glutathione metabolism” pathway, was related to the pressure-overloaded heart remodeling and dysfunction [24].

Five function terms were related to ion homeostasis in cells, such as “metal ion homeostasis”, “monovalent inorganic cation homeostasis” and “monovalent inorganic anion homeostasis”. Abnormal ion homeostasis is related to the process of cardiomyopathy and reperfusion injury after myocardial ischemia, and often occurs in patients with various heart diseases [25, 26]. Among ions, K^+^, Na^+^ and Ca^2+^ were related to core genes, since core genes could be enriched in K^+^, Na^+^, Ca^2+^-related functions. For example, “potassium ion homeostasis”, “potassium ion transmembrane transporter activity” and other functions were closely related to the steady state and transport of potassium ions. Adenosine triphosphate-sensitive potassium channels (KATP) exist on the cell surface and mitochondrial membrane of cardiomyocytes, and can adapt electrical activity to metabolic challenges, thereby maintaining the normal biological functions of myocytes [27]. “calcium: cation antiporter activity “ and “sodium ion transmembrane transporter activity” were closely related to the transportation and homeostasis of Na^+^ and Ca^2+^. Studies have found that changes in intracellular Ca^2+^ homeostasis and late Na^+^ current increased the possibility of early depolarization and delayed depolarization, which caused arrhythmia in diseased cardiomyocytes [28].

#### Literature confirmation

Among the 13 core genes, 7 genes (containing one known disease gene) have been confirmed to be related to cardiomyopathy or other heart diseases by literature.

TNNI3K was a confirmed pathogenic gene of DCM that has been implicated in various cardiac phenotypes and diseases [29, 30]. Na^+^-K^+^-2Cl^-^ cotransporter 1 (NKCC1), encoded by gene SLC12A1, and Na^+^-K^+^-2Cl^-^ cotransporter 2 (NKCC2), encoded by gene SLC12A2, were two variants or isoforms of Na-K-2Cl-cotransporter (NKCC), which was one of the most important sodium transport mechanisms that could cause the intracellular sodium concentration to increase. The increase in intracellular Na^+^ and Ca^2+^ concentration transduced nuclear signals, and triggered cardiac remodeling and hypertrophy [31]. Studies have shown that SLC12A6 is more specifically present in cardiomyocytes, vascular smooth muscle cells and various neurons [32, 33]. The genes RRM1 and RRM2 encoded two subunit proteins of ribonucleotide reductase (RNR), and RRM2B encoded the small subunit of p53-inducible RNR. The increase of RNR and/or dATP pools in heart cells could significantly alter the cycle of actin-myosin bridges, thereby enhancing the contractile function of patients with heart failure [34, 35].

Although 6 core genes have not been reported to be related to cardiomyopathy, they were enriched in the pathways and functions related to cardiomyopathy. The role of these 6 core genes in cardiomyopathy is worthy of further study.

#### Classification efficiency

To further reveal the relationship between the core genes and cardiomyopathy, the expression values of the core genes were used as the classification features to classify samples of different states (DCM_NF, ICM_NF, or DCM_ICM) in the cardiomyopathy expression data (GSE116250). The results showed that the core genes had good classification performance for normal and DCM samples (AUC = 0.996), ICM and normal samples (AUC = 0.989), and for ICM and DCM samples (AUC = 0.708).

**Fig. 6:**
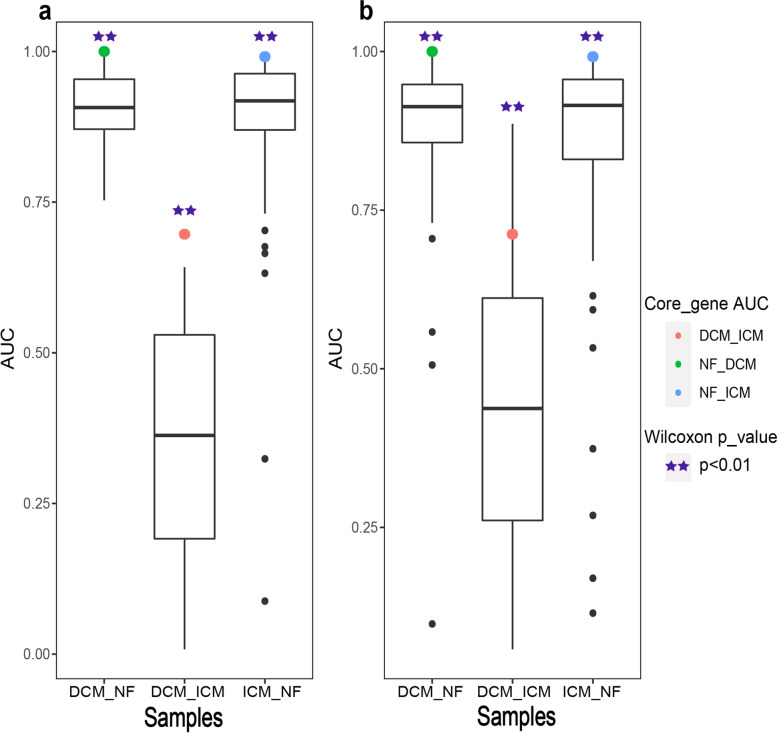
AUC values of core genes and two random sets. **a** AUC value distribution of the first set of random genes. **b** AUC value distribution of the second set of random genes. The box plots represent the AUC values of random sets classifying DCM_NF, DCM_ICM and ICM_NF samples, and the green, red and blue marks are the AUC values of core genes, respectively. The purple asterisk indicates the degree of difference between the AUC value of the core genes and the AUC values of the random sets

In addition, two sets of random genes were selected for comparison to verify the classification efficiency of core genes. The first set of random genes was comprised of genes with the same number as the core genes randomly selected from 39 candidate genes after removing the core genes. The second set contained 13 randomly selected genes with the same number of differentially and non-differentially expressed genes as the core genes. The expression values of genes in the random gene sets were used as classification features to classify samples of different states (DCM_NF, DCM_ICM or ICM_NF). The randomization was performed 100 times. The difference between the AUC value of the core genes and the AUC values of random gene sets were tested by Wilcoxon Signed Rank Test. The results demonstrated significant differences between the core genes and the two random gene sets (*p* < 0.01, Fig. 6). And the classification efficiency of core genes was significantly better than that of random genes.

To further verify the performance of the core genes in classifying samples of different states, two other publicly published independent microarray datasets GSE21610 (8 normal samples, 42 DCM samples, and 18 ICM samples) and GSE1145 (15 DCM samples, 11 ICM samples and 11 NF samples) from the Gene Expression Omnibus (GEO) database were used. The Support vector machine (SVM) algorithm was applied to these datasets, respectively. It was demonstrated that the core genes could accurately classify samples of different states in GSE21610 (AUC > 0.70) and GSE1145 (AUC > 0.80).

The above results showed that core genes could not only efficiently distinguish between different samples, but also correctly classify samples of other expression profiles. These core genes were expected to become markers for DCM and ICM.

## Discussion

Cardiomyopathy is a type of myocardial disease with abnormal heart structure and myocardial function caused by different causes. DCM and ICM are two common types of cardiomyopathy with similar clinical manifestations, and difficult to distinguish [36, 37]. Here, an integrated method was proposed to identify cardiomyopathy-related core genes in a human metabolic network using cardiomyopathy-related expression data. Three groups of initial modules were determined from the reconstructed human metabolic network. Furthermore, three candidate modules with significant differences by permutation tests were selected.

One of them was identified as a cardiomyopathy risk module, which was able to distinguish between DCM_NF samples as well as DCM_ICM samples. And 13 core genes closely related to cardiomyopathy were identified. They could effectively distinguish between samples of different states (DCM_NF, ICM_NF or DCM_ICM) and were enriched in pathways and functions related to cardiomyopathy.

Generally speaking, network modules are selected with a relatively large number of nodes, such as module with at least 5 nodes [38]. To obtain more and comprehensive initial modules, we reduced the threshold to 4 nodes, which also appeared in previous studies [39]. We further selected the initial module with the number of nodes ≥3 for analysis, and the cardiomyopathy risk module and core genes finally identified remained unchanged.

The risk module showed significant differences for both DCM_NF and DCM_ICM samples. Although the risk module was not significantly different between normal and ICM samples, the core genes identified from it could distinguish ICM samples from normal ones.

Genes of the other two candidate modules (ICM-module1 and ICM-module2) were effective to separate ICM samples from normal samples, illustrating its significant differences between the normal state and the ICM state (ICM-module1 (AUC = 0.93), ICM-module2 (AUC = 0.79)).

**Fig. 7:**
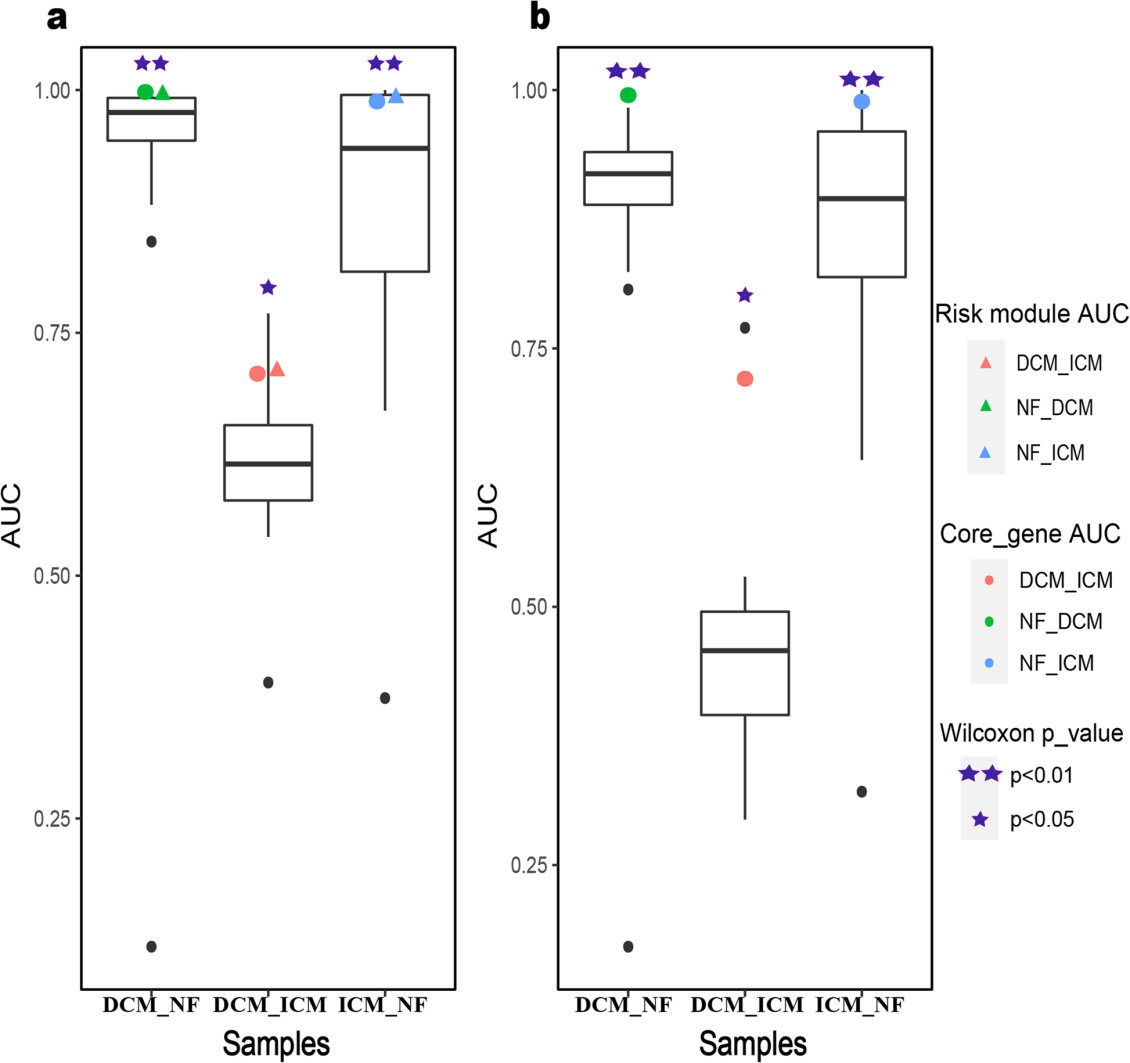
AUC values of core genes and initial modules. **a** AUC value distribution of the initial modules by the first level. **b** AUC value distribution of the initial modules by the second level. The box plots represent the AUC values of initial modules classifying DCM_NF, DCM_ICM and ICM_NF samples. The green, red and blue circles are the AUC values of core genes, while the green, red and blue triangles are the AUC values of the cardiomyopathy risk module, respectively. The purple asterisk indicates the degree of difference between the AUC value of the core genes and the AUC values of the initial modules

The classification efficiency of the core genes was compared with the classification efficiency of initial modules to prove its effectiveness. Specifically, when distinguishing ICM_NF, DCM_NF and DCM_ICM samples, the classification efficiency of core genes was compared with that of 37 ICM initial modules, 21 DCM initial modules and 8 D_I initial modules, respectively. The initial modules were compared at two levels. First, the expression values of all genes in each initial module (the cardiomyopathy risk module included) were used as classification features to classify samples in different states. Second, since the classification of more genes might obtain higher classification accuracy, for modules with more than 13 genes, 100 sets of genes with the same number of differentially and non-differentially expressed genes as the core genes were randomly selected. The expression values were used as the classification features to classify samples in different states. The average of 100 random AUC values was used for each module. The classification efficiency for ICM_NF, DCM_NF and DCM_ICM samples of core genes were significantly better than that of the initial modules of the two levels (Wilcoxon signed rank test, Fig. 7). Our identified core genes could classify samples with high accuracy.

WGCNA is a popular tool for network analysis and mining modules and hub genes based on gene co-expression [40]. To further evaluate our approach, WGCNA was performed using genes from the reconstructed metabolic network based on the GSE116250 dataset. Sixteen co-expression modules were identified using the one-step network construction function of the WGCNA R package. Genes with |gene significance| > 0.2 and |module membership| > 0.8 were selected as hub genes in each significant module. However, the core genes that we identified were not in these genes. The hub genes of these modules were respectively used as characteristics to classify samples of different states in GSE116250. The results showed that the hub genes of most modules could effectively distinguish normal and disease samples, and the hub genes of two modules could distinguish DCM and ICM samples. Our approach and WGCNA identified cardiomyopathy-related genes from different perspectives. Our approach was a systematic multi-omics integrated approach based on a metabolic network, while WGCNA conducted network analysis for co-expression between genes.

The limitation of our research was that although Recon 3 contains relatively complete metabolic reaction information, the reconstructed metabolic network was not large enough. Therefore, some known genes and DEGs were not in the network. A more comprehensive network might help to improve results, obtain more candidate modules, and identify more cardiomyopathy risk modules and core genes related to diseases.

## Conclusions

In summary, a comprehensive method based on a human metabolic network using cardiomyopathy expression data was proposed to identify cardiomyopathy-related core genes. A total of 13 core genes were identified from the cardiomyopathy risk module based on the shortest paths between known genes. These core genes could distinguish both between normal and disease samples and between DCM and ICM samples. This research will contribute to identifying potential biomarkers of cardiomyopathy and to distinguishing different types of cardiomyopathy.

## Materials and methods

### Data

#### Screening of DEGs

The expression profile by high throughput sequencing GSE116250 was downloaded from the GEO (https://www.ncbi.nlm.nih.gov/geo/), which contained 14 normal samples, 13 ICM samples, and 37 DCM samples [41]. All expressed data used in this study were processed using the following process. (1) The probes or genes with more than 50% missing values were deleted, and the remaining missing values were filled with the k-Nearest Neighbor method using the knnImputation function in R package “DMwR”. Specifically, for each missing value, its k nearest expression values were searched based on the Euclidean Distance, and the weighted average of these values was used to fill in the missing value. (2) Probes corresponding to multiple genes were deleted. (3) For multiple probes corresponding to the same gene, the average expression value of these probes was used as the expression value of the gene.

The SAM algorithm was used to find the DEGs between ICM and normal samples, between DCM and normal samples, and between ICM and DCM samples, through the R package “samr”. Finally, 1802 DEGs between DCM and normal (indicated as DCM_NF in this paper) samples (DCM_NF DEGs), 3253 DEGs between ICM and normal (indicated as ICM_NF) samples (ICM_NF DEGs), and 358 DEGs between DCM and ICM (indicated as DCM_ICM) samples (DCM_ICM DEGs) with |log2(FC)| > 1 and FDR adjusted *p*-value < 0.05 were obtained.

#### Metabolic network reconstruction

On the basis of metabolic responses extracted from Recon 3 of the Virtual Metabolic Human Database (https://www.vmh.life) [42], a metabolic network composed of protein-coding genes (nodes) and their interactions (edges) was reconstructed. Recon 3 was created by expanding Recon 2 through the addition of new publicly available metabolomics data. The metabolic network was reconstructed by the following process. The two enzymes were thought to interact if the product of the reaction catalyzed by one enzyme was the substrate of the reaction catalyzed by the other enzyme. The genes encoding the proteins that make up the two enzymes were connected in the reconstructed network. Ubiquitous metabolites such as H_2_O, CO_2_ and ADP were excluded to avoid bias due to their extreme connections. The reconstructed metabolic network contained 3105 nodes (containing 257 ICM_NF DEGs, 141 DCM_NF DEGs, and 34 DCM_ICM DEGs) and 85,880 edges.

### Mining the initial modules

Subsequently, the metabolic network was visualized with the help of Cytoscape software (version 3.7.0). In addition, the MCODE (version 1.6.1) plug-in in Cytoscape software was used to explore important modules in the metabolic network [43]. The advanced options were set to degree cutoff = 2, K-Core = 3, and node score cutoff = 0.2. The modules containing DEGs were screened as initial modules.

### Detection of candidate modules

Candidate modules with significant differences were detected from initial modules using two steps.

*W* was evaluated by the difference between the average of absolute values of Pearson correlation coefficients *H* and *H*^′^ for samples of different states (DCM_NF, ICM_NF, or DCM_ICM).

$$W\left(M\right)=\left|H-H'\right|$$

where *H* and *H′* were calculated according to expression values for all gene pairs.

$$H=\frac{1}{C_g^2}\sum_{j=1}^{C_g^2}\left| Pearson\left({X}_j,{Y}_j\right)\right|=\frac{1}{C_g^2}\sum_{j=1}^{C_g^2}\left|\frac{\sum_{i=1}^n\left({X}_{ji}-{\overline{X}}_j\right)\left({Y}_{ji}-{\overline{Y}}_j\right)}{\sqrt{\sum_{i=1}^n{\left({X}_{ji}-{\overline{X}}_j\right)}^2}\sqrt{\sum_{i=1}^n{\left({Y}_{ji}-{\overline{Y}}_j\right)}^2}}\right|$$

$${H}^{\prime }=\frac{1}{C_g^2}\sum_{j=1}^{C_g^2}\left| Pearson\left({X}_j^{\prime },{Y}_j^{\prime}\right)\right|=\frac{1}{C_g^2}\sum_{j=1}^{C_g^2}\left|\frac{\sum_{i=1}^m\left({X}_{ji}^{\prime }-{\overline{X}}_j^{\prime}\right)\left({Y}_{ji}^{\prime }-{\overline{Y}}_j^{\prime}\right)}{\sqrt{\sum_{i=1}^m{\left({X}_{ji}^{\prime }-{\overline{X}}_j^{\prime}\right)}^2}\sqrt{\sum_{i=1}^m{\left({Y}_{ji}^{\prime }-{\overline{Y}}_j^{\prime}\right)}^2}}\right|$$

${C}_g^2$ is the number of all gene pairs. *n* and *m* are the number of samples of different states (DCM_NF, ICM_NF, or DCM_ICM), and *X*_*j*_, *Y*_*j*_ and ${X}_j^{\prime },{Y}_j^{\prime }$ are the expression values of the j-th gene pair in two different states, ${X}_{ji},{X}_{ji}^{\prime }$ and ${Y}_{ji},{Y}_{ji}^{\prime }$ are the expression values of the j-th gene pair in the i-th sample, and ${\overline{X}}_j,{\overline{X}}_j^{\prime }$ and ${\overline{Y}}_j,{\overline{Y}}_j^{\prime }$ are their average expression value, respectively.

Second, permutation tests were performed on the DCM, ICM and D_I initial modules to screen the modules with significant differences. The null hypothesis was that the initial modules had no difference between different states (DCM_NF, ICM_NF, or DCM_ICM). From the reconstructed metabolic network, 1000 degree-conserved random modules (the same degree of nodes as the initial module) and 1000 size-conserved random modules (the same number of nodes as the initial module) were constructed for each initial module. The Pearson difference scores of every random module (degree-conserved and size-conserved) were calculated and compared with the score of the corresponding initial module, respectively. For each way of randomization, the *p* values of initial modules were defined as follows.

$$p=1-\frac{t}{1000}$$

where *t* is the number of random modules whose Pearson difference score is less than that of the initial module.

For initial modules with *p* value < 0.05, the null hypothesis should be rejected, so they were significantly different between different states. The initial modules that were significant in both cases (degree-conserved and size-conserved) were retained as candidate modules (both *p* values < 0.05).

The performance of candidate modules in classifying samples of different states can further reveal their relationship with cardiomyopathy. A SVM classifier was constructed to classify samples of different states (DCM_NF, ICM_NF, DCM_ICM) with the expression values of all genes in each module as features. The kernel function of SVM was set to “radial”. The performance was evaluated using leave-one-out cross validation (LOOCV). LOOCV draws one sample at a time as the test set, and the rest as the training set. Then the receiver operating characteristic (ROC) curve was drawn, and the AUC was calculated to measure the classification performance according to the classification results of the test sets.

### Identification of cardiomyopathy risk modules

Markov random field (MRF) refers to the random field with Markov characteristics, which is often used to build mathematical models to identify protein interaction subnetworks [12, 13]. In our study, an MRF model was used to evaluate the expression difference considering both DEGs and non-DEGs in a candidate module by module score based on MRF (MRFms).

For a candidate module *M* with *g* genes, it was assumed that the expression difference *E* = (*E*_1_, …, *E*_*g*_) between samples of different states formed an MRF. According to the properties of Markov random fields, the expression difference of gene g depends on the difference value of its one-step neighbor genes. Gibbs distribution was employed to specify the joint probability of *E*:

$$P(E)=\frac{1}{K}{e}^{-\frac{1}{T}F(E)}$$

where *K* is a constant that guarantees the probability sum to be 1, *T* is a temperature parameter controlling the distribution sharpness, and

$$F(E)=-\frac{1}{\sqrt{g}}\sum_{i\in {G}_1}{E}_i+\frac{1}{b}\sum_{i,j\in {G}_2}{\left(\frac{E_i}{\sqrt{d_i}}-\frac{E_j}{\sqrt{d_j}}\right)}^2 MI\left(i,j\right)$$

Based on our previous study [44] and the calculation process in [45], the MRFms for module M incorporating Mutual Information (MI) was defined as

$$MRFms(M)=\frac{1}{\sqrt{g}}\sum_{u\in {G}_1}{E}_u-\frac{1}{b}\sum_{v,z\in {G}_2}{\left(\frac{E_v}{\sqrt{d_v}}-\frac{E_z}{\sqrt{d_z}}\right)}^2 MI\left(v,z\right)$$

where *b* is the number of edges, *G*_1_ and *G*_2_ are the set of DEGs and non-DEGs in the module; *E*_*u*_, *E*_*v*_ and *E*_*z*_ are the expression differences of genes *u*, *v* and *z* between different states (DCM_NF, ICM_NF, or DCM_ICM), and *d*_*v*_ and *d*_*z*_ are the degrees of genes *v* and *z* in the network, respectively. *MI*(*v*, *z*) is the mutual information of genes *v* and *z*.

Then the same permutation test as in the previous step was used to screen out cardiomyopathy risk modules. Finally, the modules that were significant in both cases (degree-conserved and size-conserved random modules) were identified as cardiomyopathy risk modules (both *p* values < 0.05).

### Identification of core genes

According to the connection between genes and known genes in the network, core genes were further screened in the cardiomyopathy risk modules. From the Online Mendelian Inheritance in Man database [46], 43 known genes of cardiomyopathy were extracted, and 7 of them (PPCS, RAF1, TNNI3K, ABCC9, EYA4, SDHA, and TTN) were in the metabolic network. Then, the shortest paths between known gene pairs were searched, and genes in cardiomyopathy risk modules that appeared on these shortest paths were selected as candidate genes. The number of known gene pairs linked by gene *x* via these shortest paths *B*(*x*) was counted as follows.

$$B(x)=\sum_{s\ne x\ne t}{\sigma}_{st}(x)$$

$${\sigma}_{st}(x)=\left\{\begin{array}{c}1\\ {}0\end{array}\genfrac{}{}{0pt}{}{\ if\ G\left(s,t\right)=G\left(s,x\right)+G\left(x,t\right)}{otherwise}\right.$$

where *G (s, t)* is the length of the shortest path between two nodes *s* and *t. s* and *t* are known genes for cardiomyopathy in the metabolic network. *σ*_*st*_(*x*) is a variable that indicates whether any shortest path between nodes *s* and *t* passes through node *x*. If so, it is 1, otherwise it is 0.

Genes linked more known gene pairs (top upper quartile) via shortest paths were identified as core genes.

$$C=\left\{x\left|{\mathit{\operatorname{rank}}}_x\in \left[1,\left\lfloor 0.25n\right\rfloor \right],{\mathit{\operatorname{rank}}}_x=\mathit{\operatorname{rank}}\left(B(x), in\ D\right)\right.\right\}$$

where *D* is the set of *B(x)* for all *x*, *rank*_*x*_ is the rank of *B(x)* when ranking all *B(x)*s in set *D* in descending order, *n* is the number of candidate genes.

### Evaluation of core genes

In order to reflect the association of core genes with cardiomyopathy, they were analyzed from three aspects: literature verification, enrichment analysis and classification performance. Literature verification was conducted by searching literature showing the relationship between core genes and cardiomyopathy in the PubMed database (https://www.ncbi.nlm.nih.gov/pubmed). Enrichr was used for GO functional annotation and KEGG pathway enrichment of core genes [47]. The PubMed database was also used to validate the association of significantly enriched functional classes and pathways (FDR adjusted *p* < 0.05) with the disease. The expression values of the core genes were further used as features to classify samples of different states (DCM_NF, ICM_NF, or DCM_ICM) in the expression profile and independent microarray datasets. The classification performance of the core genes and of random gene sets was compared to further evaluate the classification performance of the core genes. Random gene sets were composed of randomly selected differentially and non-differentially expressed genes from the cardiomyopathy risk modules with the same number as the core genes.

## Data Availability

Our metabolic network data were extracted from the Virtual Metabolic Human Database (https://www.vmh.life). The Expression data were collected from the Gene Expression Omnibus (GEO). Among them, GEO’s accession IDs are GSE116250 (https://www.ncbi.nlm.nih.gov/geo/query/acc.cgi?acc=GSE116250), GSE1145 (https://www.ncbi.nlm.nih.gov/geo/query/acc.cgi?acc=GSE1145) and GSE21610 (https://www.ncbi.nlm.nih.gov/geo/query/acc.cgi?acc=GSE21610. All of the above data is publicly available. The code files for main steps of this study are available at https://github.com/wendyliwan/Identification-of-cardiomyopathy-related-core-genes.

## Abbreviations

DCM: Dilated cardiomyopathy
ICM: Ischemic cardiomyopathy
DEGs: Differentially expressed genes
SAM: Significant Analysis of Microarray
DCM_NF: The two states of DCM and normal
ICM_NF: The two states of ICM and normal
DCM_ICM: The two states of DCM and ICM
D_I modules: The modules containing DEGs of DCM_ICM
DCM modules: The modules containing DEGs of DCM_NF
ICM modules: The modules containing DEGs of ICM_NF
MRF: Markov random field
MRFms: Module score based on MRF
RNR: Ribonucleotide reductase
GEO: Gene Expression Omnibus
SVM: Support vector machine
MCODE: Molecular Complexity Detection
LOOCV: Leave-one-out cross validation
GO: Gene Ontology
KEGG: Kyoto Encyclopedia of Genes and Genomes
AUC: Area under the curve
MI: Mutual Information
